# On-Top Osteotomy of the Phalanx Base Combined With Modified Bilhaut: Cloquet Procedure for Atypical Radial Polydactyly

**DOI:** 10.7759/cureus.53285

**Published:** 2024-01-31

**Authors:** Akira Kodama, Shigeki Ishibashi, Masaru Munemori, Kentarou Tsuji, Nobuo Adachi

**Affiliations:** 1 Orthopedic Surgery, Graduate School of Biomedical and Health Sciences, Hiroshima University, Hiroshima, JPN

**Keywords:** congenital hand, radial polydactyly, triphalangeal thumb, on-top osteotomy, modified bilhaut–cloquet procedure, duplicated thumb

## Abstract

In this report, we present the combination of on-top plasty with a modified Bilhaut-Cloquet procedure for treating atypical radial polydactyly with duplication at the metacarpophalangeal (MP) joint and triphalangism of the radial and ulnar phalanges, hypoplastic middle phalanx of the radial thumb, and hypoplastic phalanx base of the ulnar thumb. To preserve the stable MP and interphalangeal joints of the radial and ulnar thumbs, respectively, on-top plasty involved osteotomizing the middle phalanx and transferring the distal end of the middle phalanx of the ulnar finger to the phalanx base of the radial thumb. A modified Bilhaut-Cloquet procedure was used to combine the tips and nails of both thumbs. Twelve months postoperatively, good joint alignment and thumb tip appearance were achieved. On-top plasties effectively combined the desirable parts of both thumbs. The modified Bilhaut-Cloquet technique is particularly well-suited for atypical cases, such as the present case.

## Introduction

Radial polydactyly is a common condition of congenital hand deformity. However, the clinical presentation of radial polydactyly differs greatly among cases, and surgical techniques have been adapted accordingly. Surgical techniques range from simple removal of the hypoplastic thumb to the creation of a new thumb by combining desirable parts using osteotomy, on-top plasty [1-3], or the Bilhaut-Cloquet technique. The on-top osteotomy is a method of digit migration involving the transposition of the distal segments of one digit onto the proximal segments of another, utilizing a neurovascular pedicle. The Bilhaut-Cloquet procedure consists of the coaptation of bone, soft tissue, and nail tissue after resection of the central segment of the duplicated thumb. Furthermore, modified methods for achieving better cosmetic outcomes have been reported [4,5]. These procedures can result in the formation of a normal-sized thumb with a stable interphalangeal joint, effectively combining the two thumbs.　

Herein, we report a case of a patient with atypical radial polydactyly with duplication at the metacarpophalangeal (MP) joint, triphalangism of both the radial and ulnar phalanges, hypoplastic middle phalanx of the radial thumb, and hypoplastic phalanx base of the ulnar thumb treated using on-top plasty combined with a modified Bilhaut-Cloquet procedure.

## Case presentation

**Figure 1:**
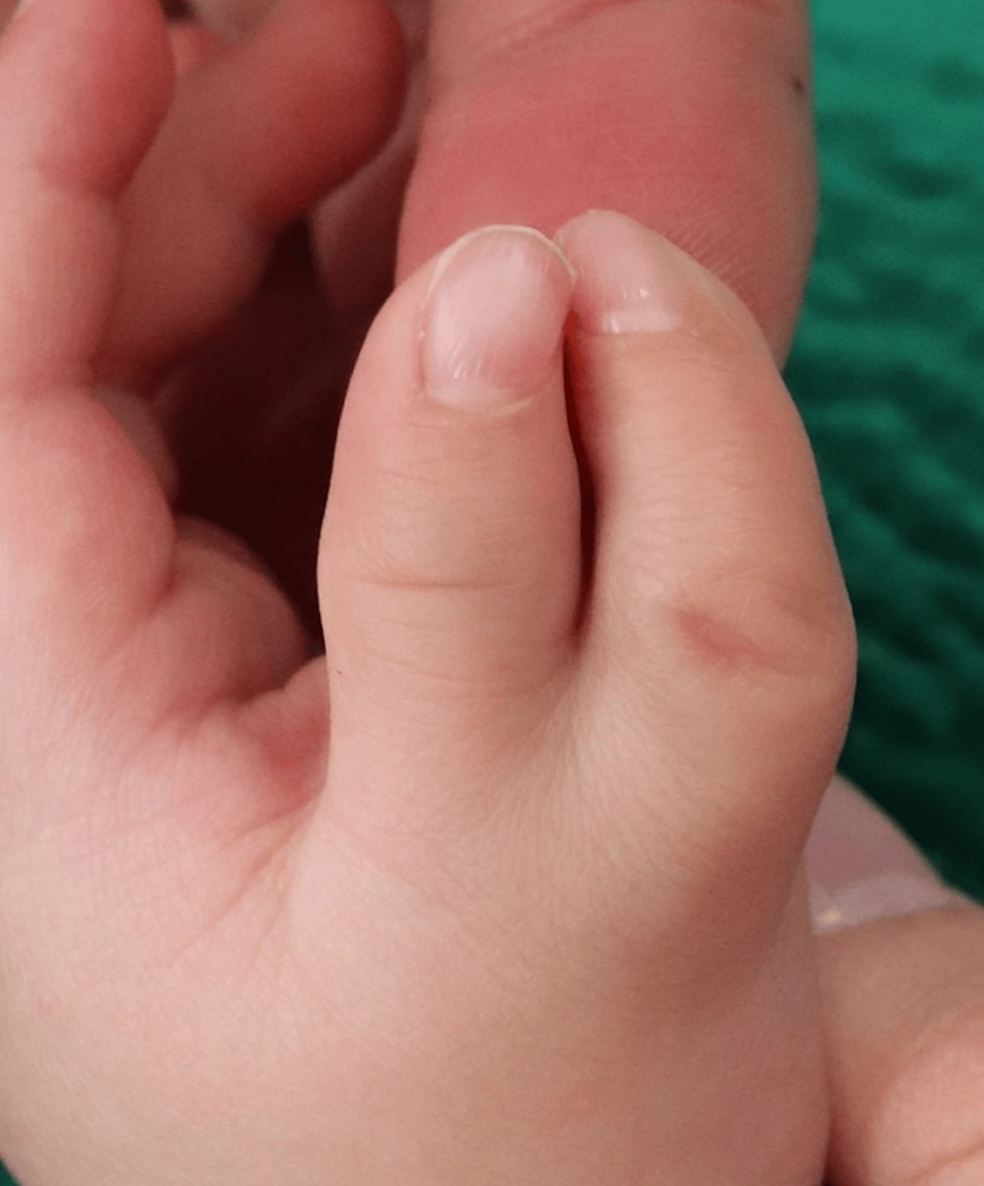
Appearance of polydactyly of the radial and ulnar triphalangeal thumbs

A 12-month-old male patient presented to our institution with left radial polydactyly duplicated at the MP joint with radial and ulnar triphalangeal thumbs (Wassel type VII). The general examination revealed no abnormal findings, except contralateral radial polydactyly with a hypoplastic accessory thumb on the radial side of the MP joint. The patient had no family history of abnormalities. A physical examination revealed nail width of 4 mm on the radial side, 6 mm on the ulnar affected side, and 9 mm on the healthy side; the nails curved toward each other, and the duplicated thumbs were both hypoplastic. The circumference measurements of the interphalangeal (IP) joint were 24 mm for the radial thumb, 24 mm for the ulnar thumb, and 30 mm for the right thumb (Figure 1).

**Figure 2:**
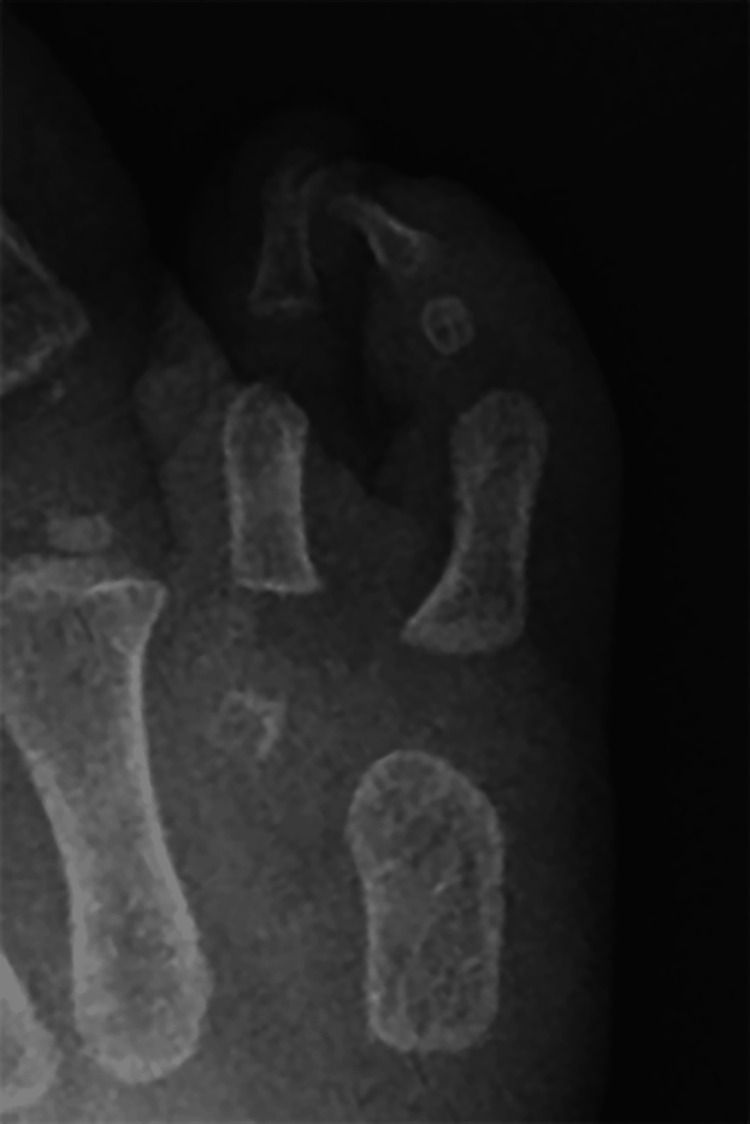
Radiographic image obtained before surgery

Radiographic examination revealed triphalangism of the radial and ulnar thumbs, with a hypoplastic extra phalanx of the radial thumb between the proximal and distal phalanx and a hypoplastic extra phalanx of the ulnar thumb at the proximal of the proximal phalanx (Figure 2).

**Figure 3:**
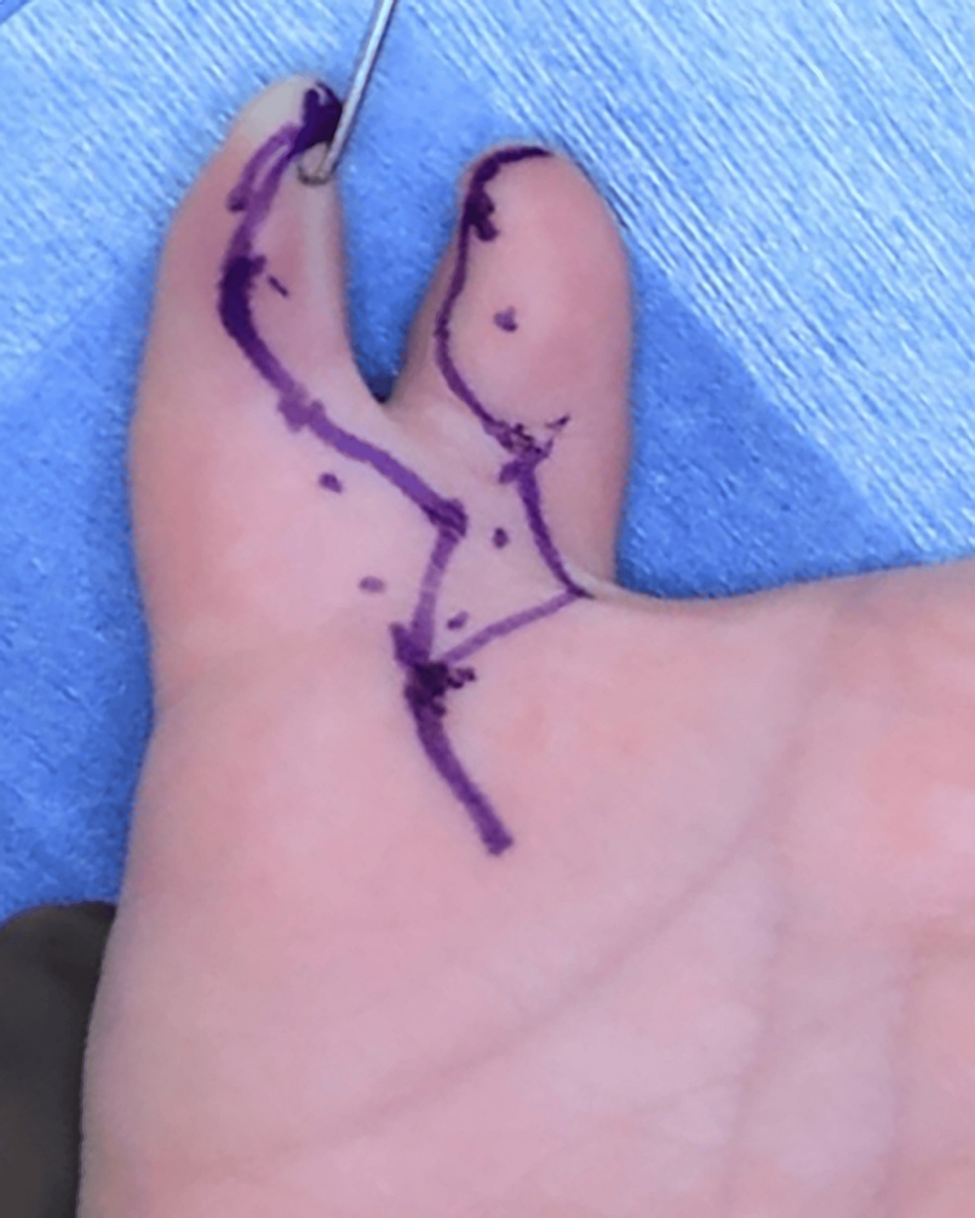
Pre-operative photograph to mark the skin incision (Palmar side)

**Figure 4:**
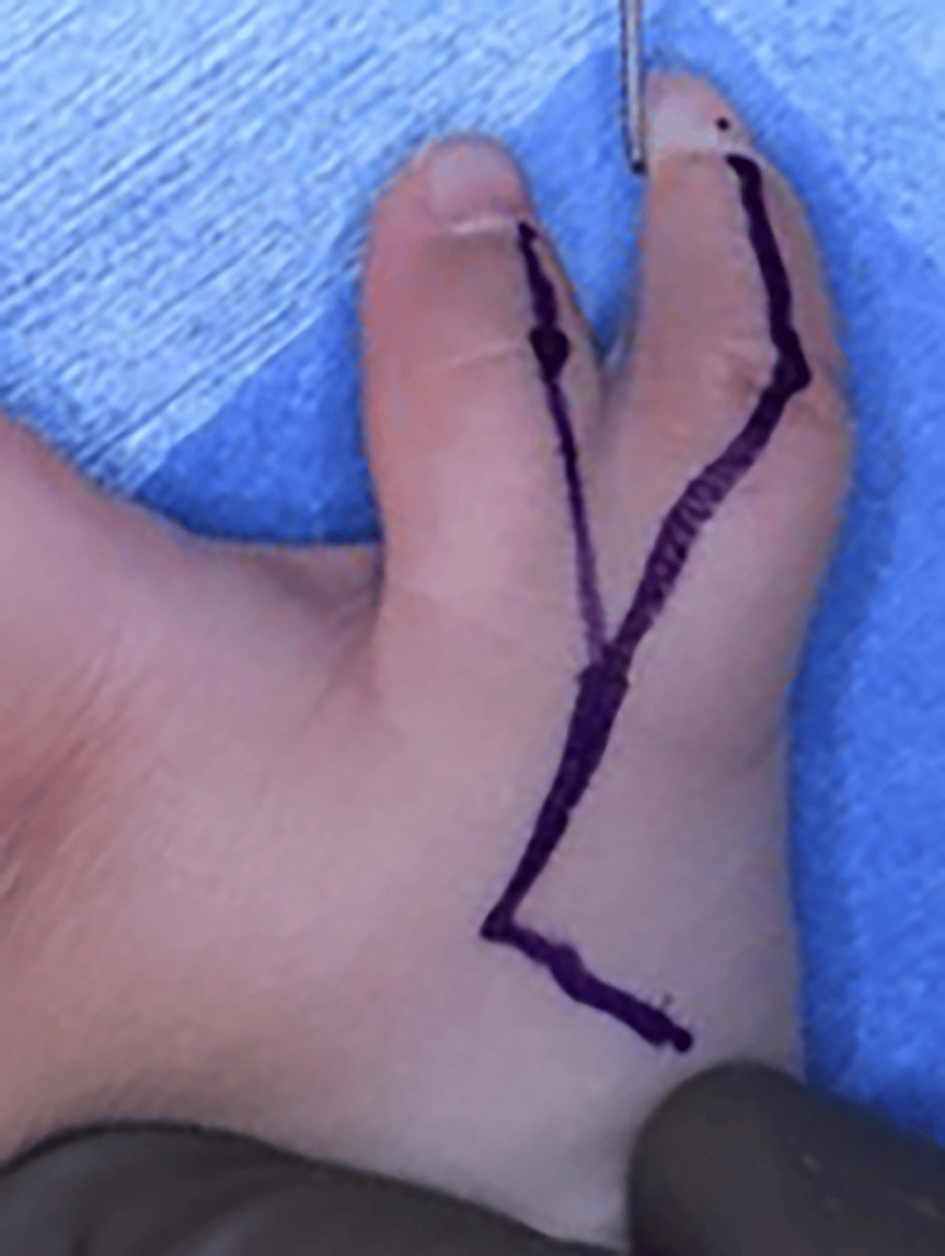
Pre-operative photograph to mark the skin incision (Dorsal side)

During surgery, we applied a zigzag skin incision from the MP joint to the thumb tips on the dorsal and volar sides (Figures 3, 4).

**Figure 5:**
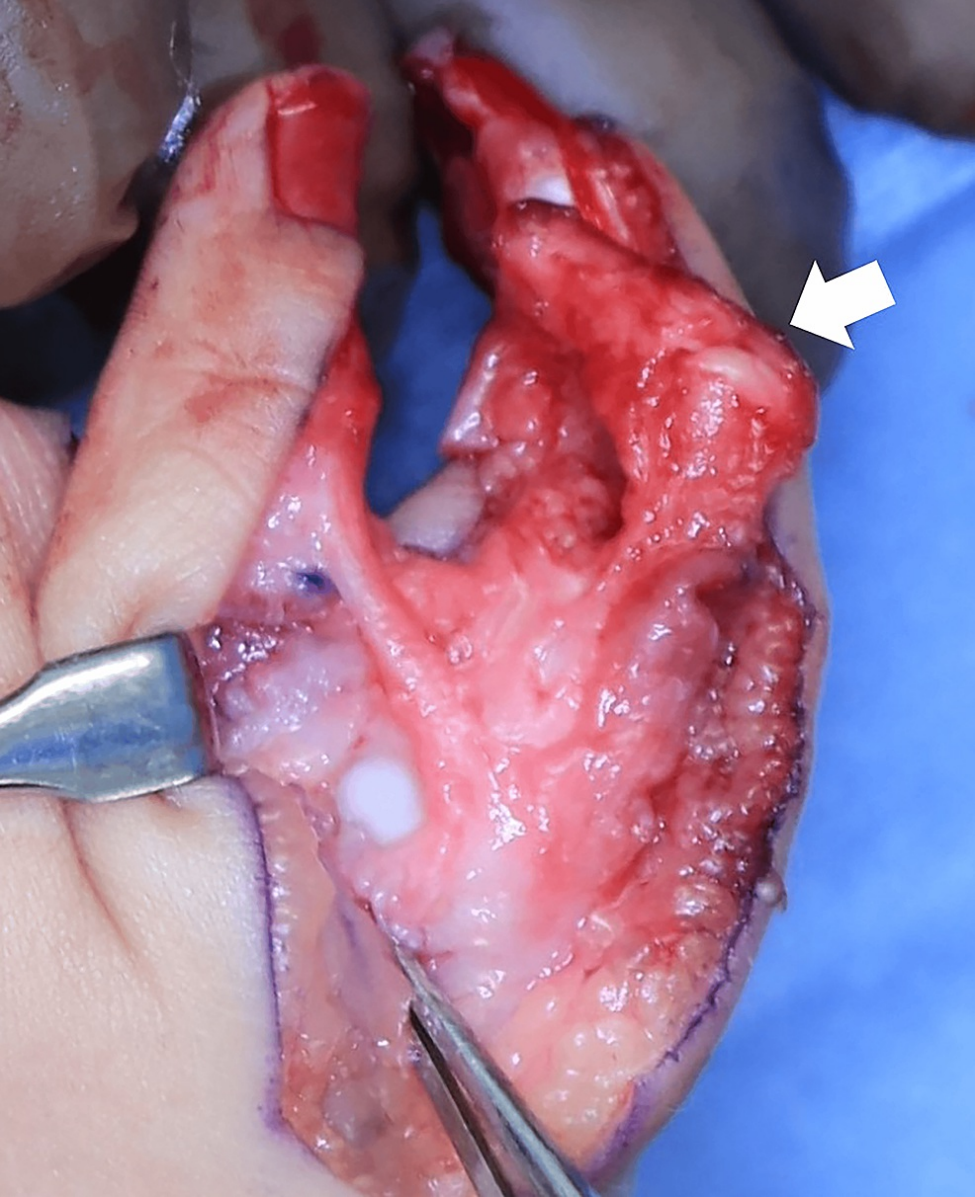
Intraoperative photograph after the excision of the proximal part of the proximal and extra phalanx of the ulnar thumb The white arrow indicates the excised part of the radial thumb, including the distal part of the proximal and extra phalanx and the ulnar 2/3 of the distal phalanx.

**Figure 6:**
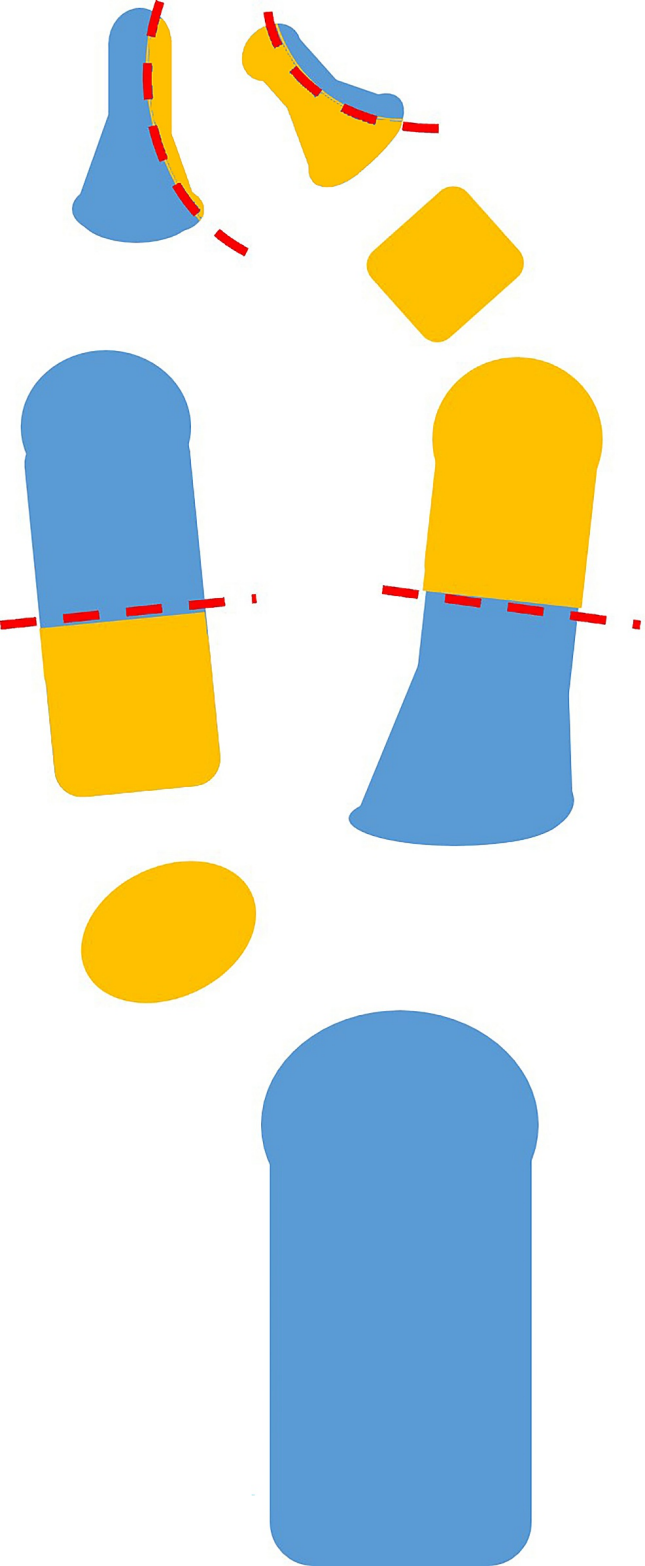
Illustrated diagram showing the excised part and the preserved part The excised part is colored orange, and the preserved part is colored blue. The red dotted lines indicate the osteotomy line.

**Figure 7:**
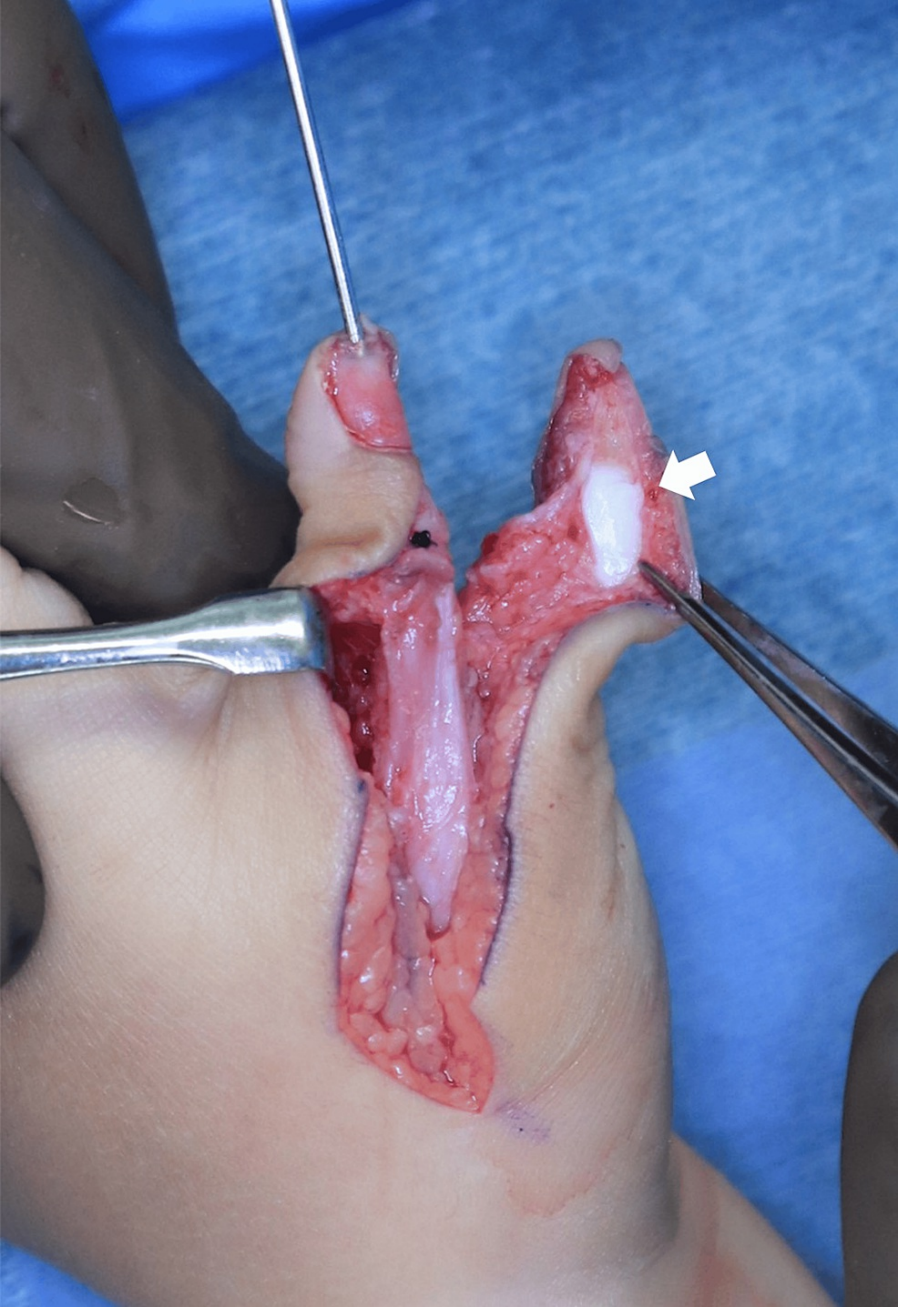
Intraoperative photograph after transferring the ulnar thumb to the radial thumb The white arrow indicates the preserved part of the distal phalanx of the radial thumb

An on-top plasty was performed to preserve the stable MP and IP joints of the radial and ulnar thumbs, respectively. Specifically, the proximal phalanx was osteotomized, and the proximal part of the proximal and extra phalanx of the ulnar thumb, the distal part of the distal and extra phalanx, and the ulnar 1/3 of the distal phalanx of the radial thumb were excised (Figures 5, 6). The distal end of the proximal phalanx of the ulnar thumb was subsequently transferred to the proximal part of the proximal phalanx of the radial thumb, and the osteotomy site involving the distal phalanx and metacarpal was fixed longitudinally with a 1.0 mm Kirschner wire (Figure 7).

**Figure 8:**
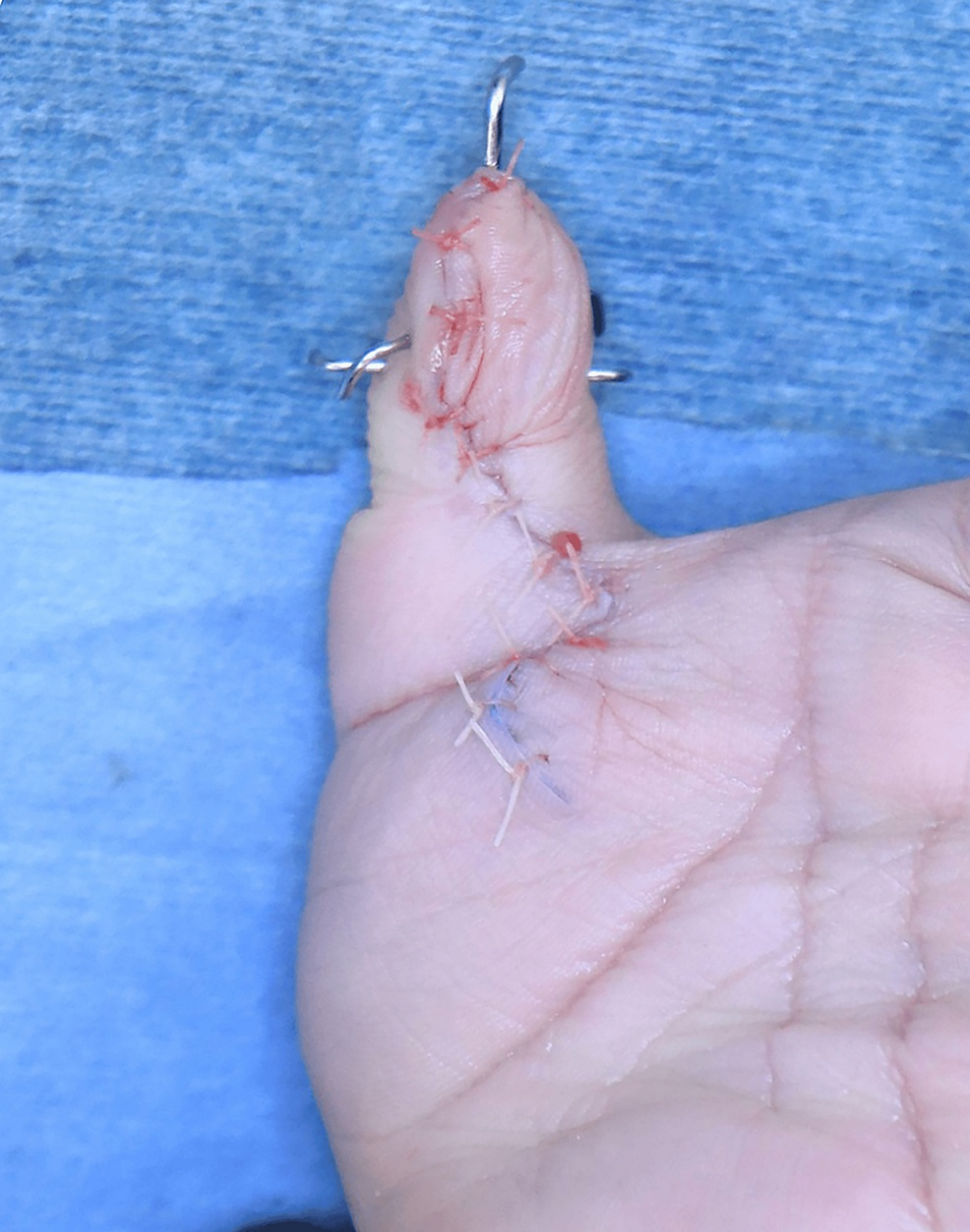
Final intraoperative appearance (Palmar side)

**Figure 9:**
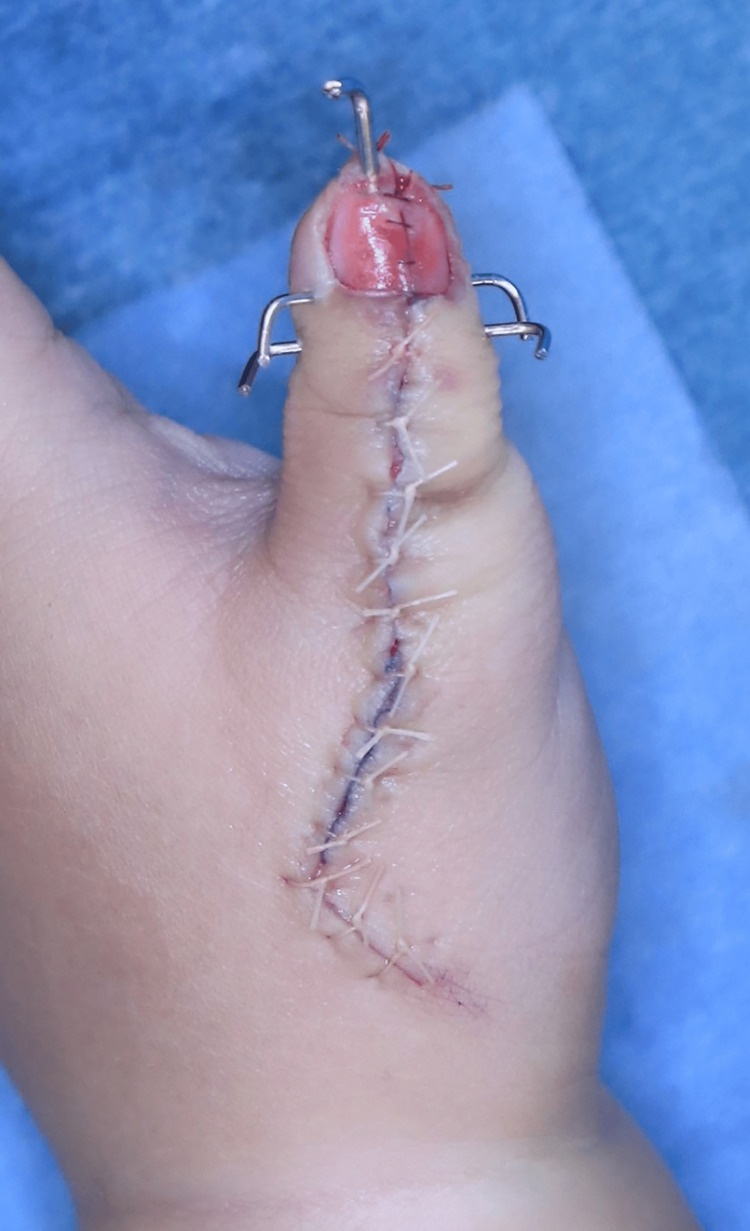
Final intraoperative appearance (Dorsal side)

**Figure 10:**
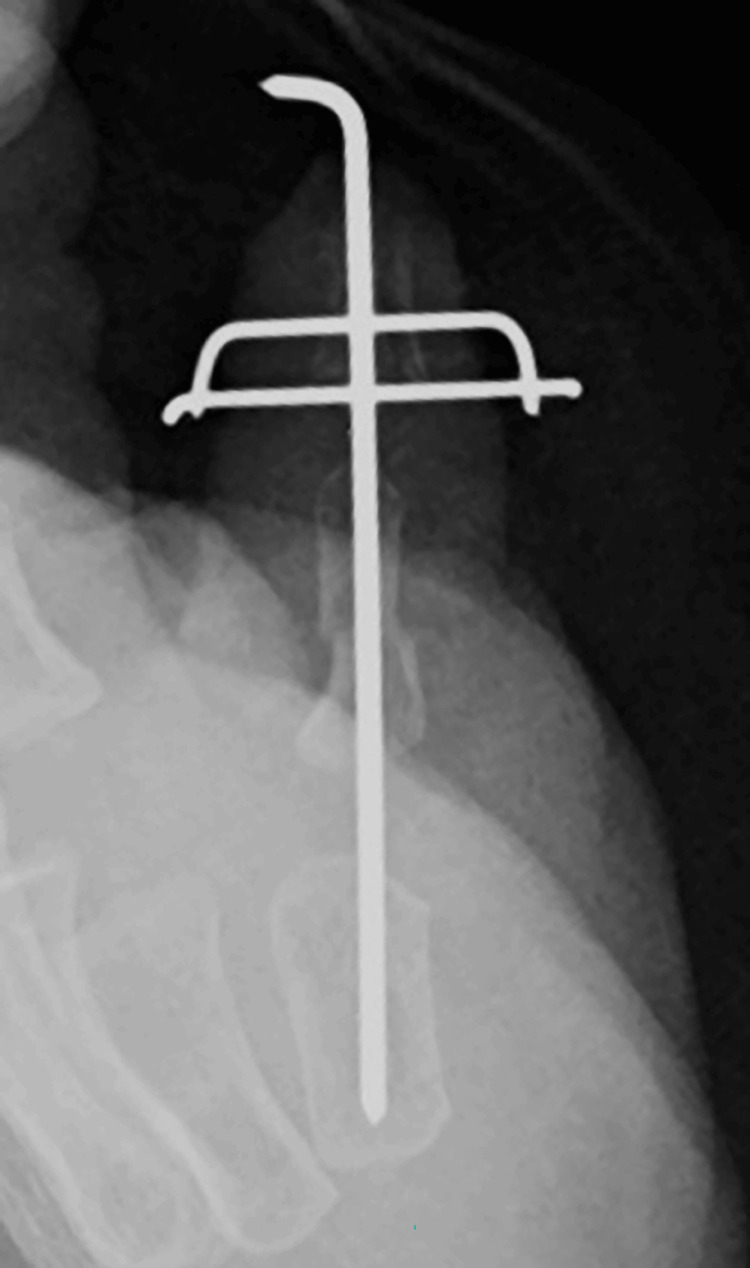
Radiographic image after the surgery

The flexor tendon of the radial thumb was transected at the bifurcation, and the extensor tendon was transected at the insertion of the distal phalanx and transferred to the extensor tendon of the ulnar thumb to balance tension. A modified Bilhaut-Cloquet procedure [4,5] was performed to combine the tips and nails of both thumbs. We created a fillet flap that included a small part of the distal phalanx to support the nail bed from the ulnar thumb. The two distal phalanxes were fixed horizontally using two 0.7-mm Kirschner wires (Figures 8-10 ).

**Figure 11:**
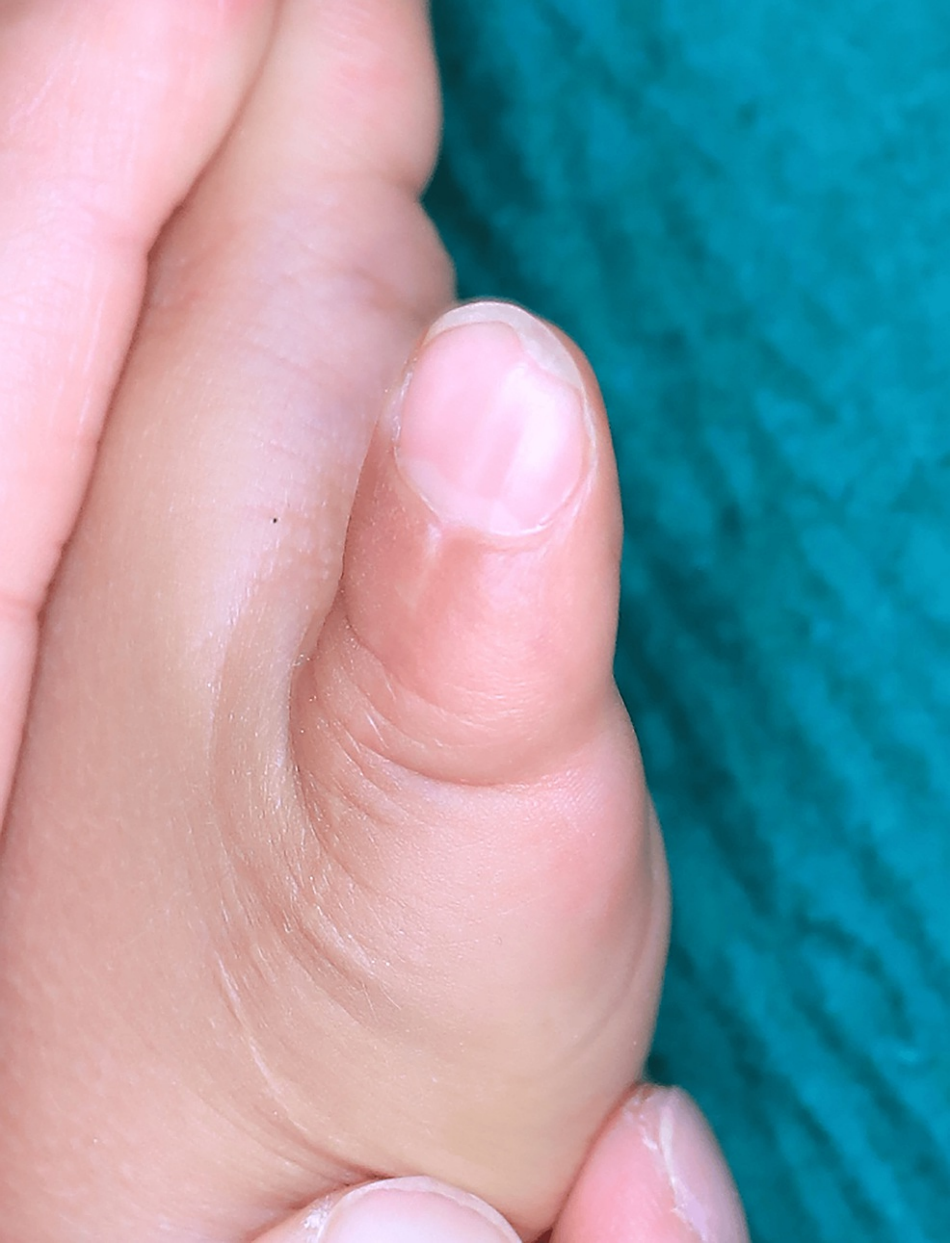
Appearance of the thumb a year after surgery

One of the nails was placed on the nail bed and secured with stitches. A mitten cast from the upper arm was worn for six weeks after surgery. The Kirschner wire was removed at the same time as the cast. Twelve months after surgery, the patient had achieved good joint alignment and thumb tip appearance, as well as useful pinch and grip functions using the reconstructed thumb. The width of the nail and circumference of the IP joint were 9 mm and 42 mm, respectively, similar to the contralateral side (Figure 11).

**Figure 12:**
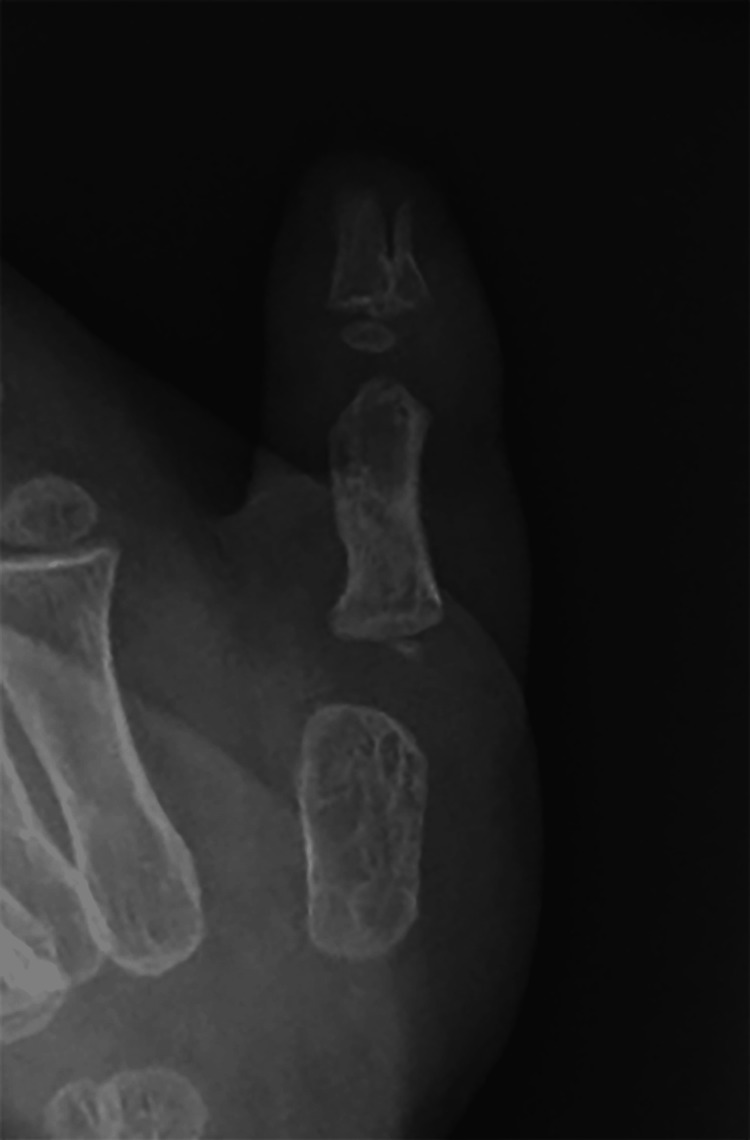
Radiographic image obtained 12 months after surgery

Radiographic examination revealed bone union of the phalanx base and distal phalanx and no remarkable angular deformity (Figure 12).

A score of 18 out of 20 points was achieved according to the postoperative outcome score for the radial polydactyly of the Japanese Society for Surgery of the Hand version [6] to assess function and appearance.

## Discussion

The present case was diagnosed as an atypical polydactyly duplicated at the MP joint with triphalangism of the radial and ulnar phalanges (Wassel type VII). Iba et al. reported two cases of atypical thumb polydactyly with duplicated metacarpals in which the hypoplastic ulnar metacarpal was prominent. For these cases, distinguishing between a duplication of the metacarpals with one of the metacarpals hypoplastic (Wassel type VI) and a duplication at the MP joint with a triphalangeal thumb (Wassel type VII) proves challenging preoperatively. However, in this patient, the operative findings revealed independently formed MP joints of the radial and ulnar thumb with complete separation of each metacarpal bone, classifying the case as Wassel type VI polydactyly [7]. The only difference between previous cases and the present case was the articulation of the ulnar thumb with the MP joint; however, the mode of occurrence was considered similar. When triphalangism is present in patients with polydactyly, one thumb may have well-structured proximal joints, whereas the other thumb may have better distal joint motion and a more aesthetically pleasing nail and pulp appearance. In such cases, an on-top osteotomy effectively combines the desirable parts of both thumbs. Several authors have described the on-top osteotomy technique for various forms of asymmetric radial polydactyly and reported favorable long-term aesthetic and functional results [1-3]. In this patient, the MP joint of the radial thumb and IP joint of the ulnar thumb were well-formed, and the IP joint of the radial thumb and MP joint of the ulnar thumb consisted of a hypoplastic extra phalanx; therefore, on-top osteotomy at the proximal phalanx was chosen to preserve the well-formed joint. Furthermore, the curved deformity of the proximal phalanx could be corrected by transferring the distal portion of the ulnarly curved radial thumb to the proximal portion of the radially curved ulnar thumb. In other words, the on-top plasty technique was instrumental in averting the apprehended zigzag deformity that might have arisen following the unilateral resection of the distal convergent polydactyly.

The Bilhaut-Cloquet procedure, used to address radial polydactyly, coaptates equal parts of the bone, soft tissue, and nail tissue following the removal of the central segment of the duplicated thumb. This technique can create a nearly normal-sized thumb and a stable IP joint. This technique is indicated for patients who have symmetrical bilateral thumbs with a nail size less than two-thirds the size of the normal contralateral thumb or smaller than that of the index finger [5]. A modified technique [4,5] can preserve the postoperative IP joint motion using an extra-articular technique. In addition, the axial rotation of the distal phalanx supporting the nail bed can be adjusted with this technique to prevent seagull deformity. Tonkin described in his review that an on-top osteotomy may be combined with a modified Bilhaut-Cloquet procedure [8]. Furthermore, the combination of on-top osteotomy and the modified Bilhaut-Cloquet technique for Wassel types IV to VII radial polydactyly was recently reported in 14 patients, demonstrating satisfactory postoperative outcomes [9]. Consequently, this technique is becoming established as part of the combination treatment for complex polydactyly. The modified Bilhaut-Cloquet technique is particularly well-suited for atypical cases, such as in the present patient, in which there is one proximal triphalangeal thumb and one distal triphalangeal thumb with bilateral hypoplastic fingernails and pulp.

## Conclusions

We reported the successful treatment of an atypical radial polydactyly with duplication at the metacarpophalangeal (MP) joint, triphalangism in both the radial and ulnar thumbs, and hypoplastic thumb elements using a combination of on-top osteotomy and a modified Bilhaut-Cloquet technique. Our results highlight the effectiveness of combining these surgical techniques in complex and atypical polydactyly cases to achieve favorable aesthetic and functional results.
